# Liver Hydatid Cyst Masquerading as a Liver Abscess

**DOI:** 10.7759/cureus.34334

**Published:** 2023-01-29

**Authors:** Hezborn M Magacha, Venkata Vedantam, Neethu Vedantam, Ashwin Jagadish

**Affiliations:** 1 Epidemiology and Biostatistics, East Tennessee State University College of Public Health, Johnson City, USA; 2 Internal Medicine, East Tennessee State University- Quillen College of Medicine, Johnson City, USA; 3 Infectious Diseases, East Tennessee State University- Quillen College of Medicine, Johnson City, USA

**Keywords:** cyst hydatid, hepatic hydatid cyst, masquerading conditions, liver abscess aspiration, incidental liver abscess

## Abstract

Hydatid cyst of the liver is a rare zoonotic disease in the United States. It is caused by Echinococcus granulosus. This disease is mainly seen among immigrants from countries where this parasite is endemic. Differential diagnoses of such lesions can include pyogenic or amebic abscesses, in addition to other benign or malignant lesions. We report the case of a 47-year-old woman who presented with symptoms of abdominal pain and was diagnosed with a hydatid cyst of the liver masquerading as a liver abscess. Microscopic and parasitological tests confirmed this diagnosis. The patient was treated and discharged without further complications during follow-up.

## Introduction

Echinococcus granulosus is the most frequent parasite that causes hydatid cysts. This infection is endemic to some parts of the world, especially in the developing world, and is very rare in the United States. Few cases have been diagnosed in patients from developing countries [1]. The risk of infection increases when handling livestock or infected dogs at home, especially in areas where parasitic infection is a public health concern, such as Africa, the Middle East, the South, and Central America. The clinical presentation of hydatid cysts depends on the localization of the cyst, which can range from nausea, vomiting, abdominal pain, hives, coughing, jaundice, and bloody stools to serious medical complications such as anaphylaxis due to a rupture into the peritoneum or chest, and death. Hydatid cysts can present with rare symptoms mimicking malignancy, and in such circumstances, the differential may be difficult. About 60% of patients infected with the parasite remain asymptomatic, and the incubation period can range from months to years. The advances in imaging have made it easier to diagnose hydatid cysts, and surgery and antimicrobials remain the most effective treatment methods [2].

In this article, we report the case of a 47-year-old immigrant woman from Mexico who presented with a hydatid cyst of the liver masquerading as a liver abscess.

## Case presentation

A 47-year-old female patient with no previous medical history presented to the emergency department with sudden onset, sharp epigastric pain that started 12 hours prior, with a level of pain ranging from seven to 10 (on a scale of 0-10) without any associated symptoms. The patient was an immigrant from Mexico who came to the United States several years ago and had no history of recent travel outside the United States and no contact with pets. On presentation, the patient’s temperature was 96.3 degrees Fahrenheit, her heart rate was 58 beats per minute, and her blood pressure was 118/63 mmHg. On examination, the patient had scleral icterus and mild tenderness in the epigastric and left upper quadrant. Significant laboratory findings included a white blood cell count of 11.1(K/µL) with an absolute neutrophil count of 64%, a lymphocyte count of 12%, an eosinophil count of 19%, a bilirubin level of 2.5 mg/dL, aspartate transaminases of 527 mg/dL, alanine transaminases of 333 mg/dL, alkaline phosphatase of 44 mg/dL, and lactate of 3.0 mmol/L.

The laboratory findings are shown in Table 1 below.

**Table 1:** Laboratory findings and reference values AST: aspartate transaminase; ALT: alkaline transaminase; BUN: blood urea nitrogen; GFR: glomerular filtration rate; pCO_2_:partial pressure of carbon dioxide; HCO3: bicarbonate

	Laboratory values	Patient values			Reference values
		Day 1	Day 2	Day 3	
White blood cell count		11.1	11.2	8.8	(3.5-10.5) K/µL
Neutrophils		64	69	68	(45-75) %
Eosinophils		19	16	10	(0-5) %
Lymphocyte		12	9	14	(20-50) %
Albumin		1.8	2.9	2.7	(3.5-5.2) g/dL
Total bilirubin		2.5	2.0	1.1	(0.3-1.2) mg/dL
Alkaline phosphatase		53	48	62	(34-104) IU/L
AST		527	408	104	(15-41) IU/L
ALT		333	260	115	(14-54) IU/L
Lactate		3.0	-	1.8	(0.5-2.0) mmol/L
Creatinine		0.58	0.54	0.41	(0.6-1.10) mg/dL
BUN		14	13	17	(6-20) mg/dL
Calcium		6.3	8.1	7.8	(8.6-10.0) mg/dL
Magnesium		1.3	1.4	1.9	(1.6-2.6) mg/dL
Total protein		3.1	4.2	4.8	(6.4-8.3) g/dL
GFR		110	112	123	(>60) ml/min/SA
Sodium		139	142	137	(136-145) mmol/L
Potassium		4.1	4.7	3.8	(3.5-5.1) mmol/L
Chloride		114	110	104	(98-107) mmol/L
Glucose (random)		162	140	80	(70-99) mg/dL
pH arterial pCO2 arterial		7.23 45	7.31 46	7.45 40	(7.35-7.45) (34-45)
HCO3		17.8	18.8	24.8	(22.0-26.0) mEq/L
Platelet count		199	250	304	(150-450) K/µL

**Figure 1:**
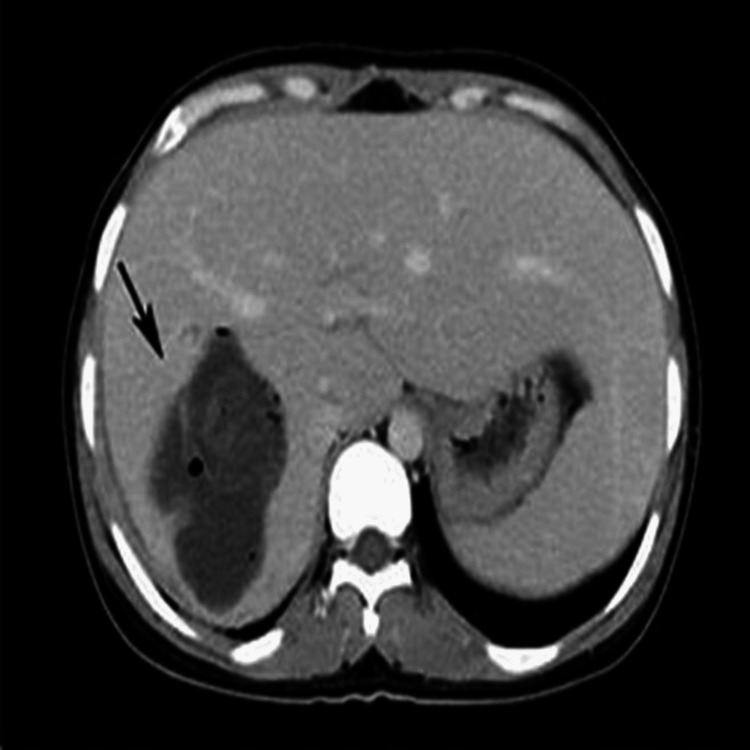
A CT scan (with contrast) of the abdomen An infected hydatid cyst of the liver in a 47-year-old woman. A contrast-enhanced CT scan shows a cystic lesion and collapsed membranes in the right lobe of the liver (black arrow).

A contrast-enhanced CT scan of the abdomen revealed a 4.5 x 2.8 cm water density lesion in the right lobe of the liver, with surrounding enhanced margins at segment six of the liver suspicious of a liver abscess or an infected cyst (Figure 1).

**Figure 2:**
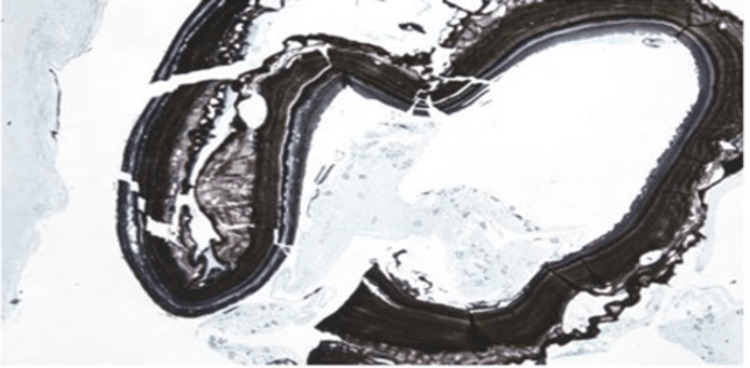
Grocott methenamine silver (GMS) stains the cystic wall

**Figure 3:**
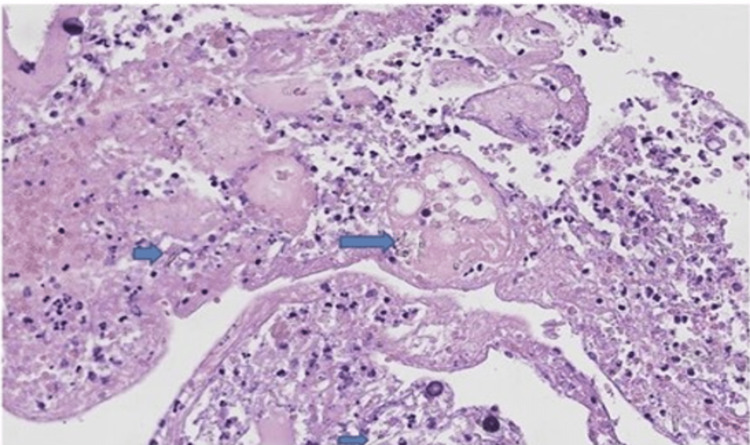
Protoscolex of Echinococcus showing hooklets (arrow)

**Figure 4:**
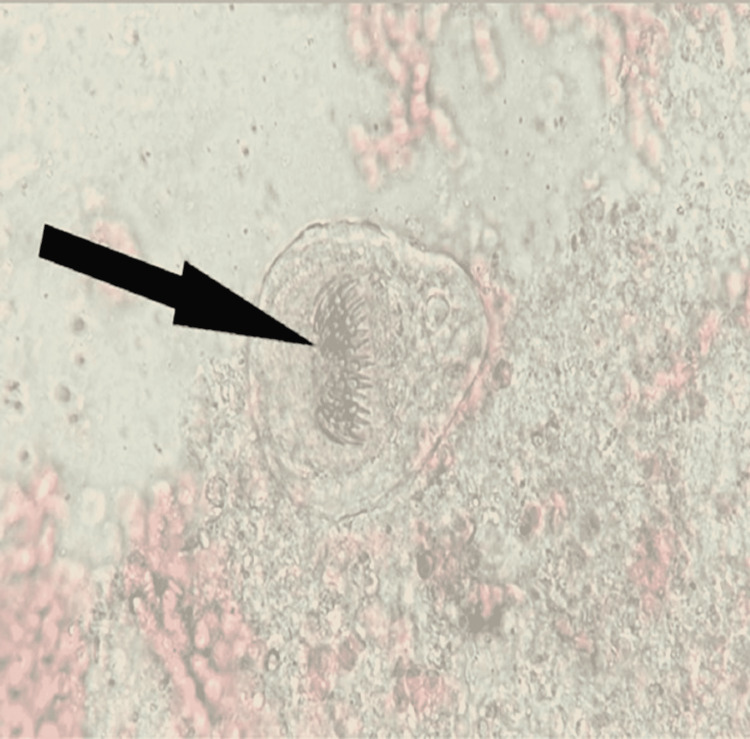
Direct microscopic examination (at 40x magnification) showing protoscoleces. Note the row of hooklets (arrow).

She was started on intravenous piperacillin-tazobactam at 3.375 g every six hours (3.375 g IV q6hr), and interventional radiology performed percutaneous aspiration and drain placement, considering the concern for abscess. The patient tolerated the procedure well and experienced no complications. Aerobic and anaerobic cultures of the fluid did not yield any organisms. The patient remained afebrile and had no associated leukocytosis after starting the antibiotics. The cytology of the drained fluid revealed mixed inflammation, degenerative debris, and background-scattered retractile hooklets with a few protoscolices. The microscopic studies of the cytological fluid demonstrated protoscolices and hooklets. These features are compatible with hydatid cysts (Figures 2-4).

The patient was started on albendazole 400 mg twice daily. The CT-scan imaging did not reveal any involvement of the other organs. Magnetic resonance imaging (MRI) of the abdomen revealed a second cyst, and the staging of the main cyst was classified as CE3B. Puncture-aspiration-injection-respiration (PAIR) was not available at our facility; therefore, the drain was removed and surgery was consulted. Echinococcal serology results were positive.

The patient underwent a laparoscopic partial hepatectomy. She was found to have scattered nodules in the peritoneum with concern for seeding, given her recent aspiration and drain placement. A biopsy of the inflammatory nodules in her abdomen revealed no viable organisms. Her postoperative course was complicated by postsurgical hemorrhage of the liver, and the patient underwent exploratory laparotomy with suturing of the bleeding and hematoma evacuation. She subsequently recovered and was discharged on oral albendazole 400 mg twice daily for six months.

## Discussion

Hydatid disease is a parasitic infection affecting humans caused by the larval stages of cestodes of the genus Echinococcus, of which Echinococcus granulosus is the most frequently encountered species. Adult parasites reside in the stomachs of dogs, which are their definitive hosts. It reproduces in the intestine and releases eggs in the feces, which are then ingested by intermediate hosts when they graze on grass contaminated by eggs. Sheep, cattle, and horses serve as intermediate hosts. Rarely can humans get infected by handling or ingesting food, water, or soil contaminated with stool from infected dogs [3]; therefore, they are referred to as aberrant intermediate hosts. When ingested, the larvae of these eggs enter the liver via the portal vein and are distributed to different organs [4]. Among all organs, the liver is the most affected (70% of the cases), followed by the lungs (20% of the cases reported), and other organs less frequently [5].

Rare cases of cysts have been identified in the brain [6] with transient neurological deficits and in the pancreas [7], which can present as obstructive jaundice.

Hydatid cysts can present with various symptoms and clinical complications, depending on the organ involved. Hydatid cysts and liver abscesses have nonspecific clinical manifestations, including right abdominal pain, fever, nausea, and vomiting, which can simulate either malignancy or cysticercosis. Non-specific clinical symptomatology makes it difficult to make a clear diagnosis based on clinical presentation alone [4].

Untreated hydatid disease can result in complications. Therefore, it is important to suspect this in all susceptible patients presenting with complaints and imaging findings consistent with hydatid disease. One such complication is cyst infection and subsequent abscess formation. Such complications are usually latent and subclinical and can present clinically with right upper quadrant pain associated with other symptoms such as fever, nausea, and vomiting [5,7]. Other complications include cyst rupture that can cause life-threatening anaphylaxis and peritonitis [8]. Suspicion of an Echinococcus cyst prior to any interventional procedure makes it possible to avoid intraoperative cyst rupture, which can lead to severe complications such as peritonitis, and when available, albendazole-based therapy reduces the risk of dissemination [9].

Together with imaging and cytological studies, other diagnostic studies include serological enzyme-linked immunosorbent assay (ELISA), Western blot, and microscopic studies. In our case, serological studies were compatible with Echinococcus, and microscopic studies and observations revealed scattered retractile hooklets with a few protoscolices present, compatible with the parasite. Microscopic observation of the parasite is a confirmatory diagnostic test for hydatid cysts [3].

Liver hydatid cysts can be treated medically or surgically using minimally invasive procedures such as PAIR. In our case, the patient was treated with albendazole and surgically treated with a laparoscopic partial hepatectomy. Surgical treatment remains the preferred choice of treatment over medical treatment for hydatid cysts, as it completely eradicates the parasite. However, in certain circumstances where there are contraindications to surgical management, such as dead cysts, multiple cysts, and unstable patient conditions, conservative methods with drug therapy combined with or without minimally invasive procedures are the recommended treatment options. Surgical management can result in several complications, including rupture of the cyst, obstruction of the biliary tree, and infections [10].

## Conclusions

This case, being rare in the United States, highlights the importance of suspecting echinococcal infections in high-risk immigrant populations, presenting with symptoms and imaging studies suggestive of liver abscess of any etiology, even years after departure from endemic areas, as this can be silent for years. Obtaining a good medical history and careful physical examination with the help of microbiological, serological, and radiological tests can help reach a diagnosis. Intraperitoneal rupture of cysts during diagnostic or therapeutic aspiration can be fatal, causing serious complications such as biliary peritonitis. Therefore, it is vital to suspect this in all high-risk patients presenting with liver abscesses, as positive serological testing prior to aspiration will mitigate the risk of life-threatening secondary peritonitis.
